# Social-demographics, health behaviors, and telomere length in the Mexican American Mano a Mano Cohort

**DOI:** 10.18632/oncotarget.19903

**Published:** 2017-08-03

**Authors:** Hua Zhao, Lixia Han, David Chang, Yuanqing Ye, Jie Shen, Carrie R. Daniel, Jian Gu, Wong-Ho Chow, Xifeng Wu

**Affiliations:** ^1^ Department of Epidemiology, The University of Texas MD Anderson Cancer Center, Houston, TX, USA

**Keywords:** telomere length, cancer risk, lifestyle factors, social context, Gerotarget

## Abstract

In the current study, we examined cross-sectional associations among social-demographics, lifestyle behaviors, and relative telomere length (RTL) in peripheral blood leukocytes, as well as longitudinal relationships among major chronic diseases, weight gain, and RTL, among 12,792 Mexican Americans aged 20 to 85 years in the Mano-A-Mano, the Mexican American Cohort. As expected, RTL was inversely correlated with age (*ρ*=-0.15, *ρ*<0.001). In the multivariate analysis, we found that RTL was positively correlated with levels of education (*ρ*=0.021), self-insurance (*ρ*=0.041), body mass index (BMI) (*ρ*<0.001), and sleeping time per day (*ρ* for trend<0.001), and RTL was inversely correlated with sitting time per day (*ρ* for trend =0.001). In longitudinal analysis, we found that longer RTL was modestly but positively associated with increased risks of overall cancer (adjusted hazard ratio (adj.HR)=1.05, 95% conference interval (95%CI)=1.02-1.09). In quartile analysis, 4^th^ quartile (longest RTL) was associated with 1.53-fold increased risk of overall cancer (adj.HR=1.53, 95%CI=1.11-2.10), compared to 1^st^ quartile (shortest RTL). RTL was reversely associated with the risk of type-2 diabetes (adj.HR=0.89, 95%CI=0.82-0.94). In quartile analysis, 4^th^ quartile (longest RTL) was associated with 48% decreased risk of typle-2 diabetes (adj.HR=0.52, 95%CI=0.32-0.70), compared to 1^st^ quartile (shortest RTL). In addition, longer RTL was a positive predictor of at least 10% weight gain (adj.HR=1.03, 95%CI=1.00-1.05). In summary, our results in Mexican Americans support the notion that telomere length is a biological mechanism by which social demographics and health behaviors “get under the skin” to affect health.

## INTRODUCTION

Telomeres are protective structures that cap the end of eukaryotic chromosomes, comprising multiple 5′-TTAGGG-3′ repeats, ending in a single-stranded overhang of the G-rich sequence [1]. As a normal cellular aging process, telomere length decreases with age. However, the speed at which telomeres shorten varies significantly among individuals. Many factors, including genetic, environmental, socio-demographics, cultural and behavioral factors [2-5], may affect the rate of telomere length shortening. Critically short telomeres may lead to chromosomal degradation, end-to-end fusion, and abnormal recombination, processes involved in disease development. On the other hand, cells with longer telomeres may favor delayed cell senescence, thus increasing the chance of developing chromosomal instability and genetic aberrations, eventually leading to carcinogenic transformation [6]. Both longer or shorter telomere length have been associated with increased risks of major age-related chronic diseases, such as cancer [7-9], type-2 diabetes [10], and cardiovascular disease [11] in epidemiological studies.

Age-related chronic diseases have also been linked to low socioeconomic status (SES) [12-17]. A common hypothesis for these associations is that chronic stress associated with social disadvantage contributes to deterioration of the body, which accelerates the decline in physiological functioning [18]. Such decline may affect leukocyte telomere length and further impact biological aging and the risk of age-related chronic diseases. Leukocyte telomere length may therefore be a link between lifestyle and environmental exposures associated with social disadvantage and age-related chronic diseases [2, 19, 20]. Maintaining a healthy lifestyle, including keeping a normal weight, regular physical activity, not smoking, and not heavy drinking, has been associated with decreased levels of oxidative stress and inflammation [20-23], and helps to prevent chronic diseases [19, 20, 24, 25]. Reduced levels of oxidative stress and inflammation have also been biologically linked to improved telomere maintenance [26-29]. However, evidence from population-based cohort studies on the relation between health behaviors and leukocyte telomere length has been inconsistent [19, 20, 30-32].

Studying the role of leukocyte telomere length in stress, biological aging, and age-related chronic diseases is particularly relevant to Mexican Americans. Mexican Americans are one of the fastest-growing populations in U.S. [33]. Three out of four Mexican Americans are either overweight or obese [34, 35]. In addition, they participate in low levels of physical activity [36], and recent surveillance data show only 44.6% met national physical activity recommendations [37]. Compared to non-Hispanic Whites, Mexican Americans tend to have lower SES, including lower income and homeownership, less education, and worse access to health care [38]. In addition, Mexican Americans are experiencing high burden of age-related chronic diseases, such as type-2 diabetes and cardiovascular diseases [39-42]. They have unique psychological, somatic, and social stress associated with acculturation [43, 44]. Studies have shown that acculturation may increase risk for stress, stress-related diseases, and poor health behaviors in Mexican Americans [45-47].

To date, the relationship among social demographics, lifestyle behaviors, and leukocyte telomere length have not been investigated in any prospective cohort study in adult Mexican Americans. Here, taking advantage of the large ongoing prospective Mano-A-Mano, the Mexican American Cohort study, we measured relative telomere length (RTL) in peripheral blood leukocytes from 12,792 cohort participants, and investigated its relationships with social demographics, lifestyle behaviors, and major chronic diseases at baseline, and with the development of cancer and type-2 diabetes at follow-up. Additionally, we studied whether RTL could predict prospective weight gain during follow-up.

## RESULTS

Of 12,792 study subjects measured for the RTL, 614 were excluded for further analysis due to questionable RTL value (outside of ±3 standard deviation of age-adjusted mean RTL). Thus, a total of 12,178 study subjects were included for further data analysis. At baseline, the mean and median ages of the population were 42 and 39 years old, respectively. The age ranged from 20 to 85 years old. The majority of study subjects were women (79.56%) and married (or living together) (77.00%) (Table 1). We used the levels of education, health insurance, home and vehicle ownership to assess SES. Only 40.60% of study subjects had at least high school education. For health insurance, 49.66%, 46.23%, and 68.55% had self, partner, and kids insurances, respectively. Home ownership was reported by 45.96% and vehicle ownership by 78.41%of the population. For immigration-related variables, the majority was born in Mexico (73.77%) and had low language acculturation (63.28%). Among Mexico-born study subjects, 29.53% arrived in the U.S. at ages younger than 20 years and 46.39% have lived in the U.S. for more than 15 years. We included 6 lifestyle behaviors in the analysis, namely, cigarette smoking, alcohol consumption, body mass index (BMI), sedentary behavior, sitting time, and sleeping time. Sedentary life style was defined based on 2008 Physical Activity Guidelines for Americans [48]. The majority of study subjects were overweight (BMI: 25-30) or obese (BMI > 30) (85.04%), never smokers (71.80%), never alcohol drinkers (66.66%), and sedentary (79.06%). Over half of study subjects had more than 2 hours sitting time per day (53.42%), and the majority of study subjects had 7 to 8 hours sleeping time per day (60.36%). The most prevalent chronic disease was hypertension (19.48%), followed by type-2 diabetes (15.27%), gallbladder disease (8.83%), asthma (3.97%), cancer (2.65%), and kidney disease (1.91%).

**Table 1:** Distribution of selected socio-demographics, health behaviors, and major diseases and RTL among 12,178 Mexican American study subjects

Variables	Number of study subjects (%)	Relative telomere length	*P* value*
**Age at enrollment**			
20-31	3,116 (25.59)	0.81	
32-39	3,104 (25.49)	0.78	
40-50	2,925 (24.02)	0.75	
>50	3,033 (24.91)	0.72	<0.001
**Gender**			
Men	2,535 (20.44)	0.76	
women	9,866 (79.56)	0.76	0.324
**Marital Status**			
Married/living as married	9,365 (77.00)	0.76	
Other	2,797 (23.00)	0.76	0.780
**Education**			
<High school	7,229 (59.40)	0.76	
At least high school	4,941 (40.60)	0.77	0.069
**Self insurance**			
Yes	5,498 (49.66)	0.77	
No	5,574 (50.34)	0.76	0.004
**Partner insurance**			
Yes	4,227 (46.23)	0.77	
No	4,917 (53.77)	0.76	0.189
**Kids insurance**			
Yes	6,502(68.55)	0.77	
No	2,983(31.45)	0.76	0.264
**Own home**			
Yes	5,025 (45.96)	0.76	
No	5,909 (54.04)	0.76	0.539
**Own car**			
Yes	8,558 (78.41)	0.76	
No	2,356 (21.59)	0.77	0.618
**Birth location**			
Mexico	8,973 (73.77)	0.76	
U.S.	3,191 (26.23)	0.76	0.221
**Years lived in U.S. (for born in Mexico only)**			
<5	1,038 (11.57)	0.75	
5-10	1,841 (20.52)	0.77	
10-15	1,930 (21.51)	0.76	
>15	4,162 (46.39)	0.77	0.255
**Age at arrival (for born in Mexico only)**			
<20	2,649 (29.53)	0.76	
20-29	3,906 (43.55)	0.77	
≥30	2,415 (26.92)	0.76	0.242
**Language acculturation**			
High	4,449 (36.72)	0.76	
Low	7,666 (63.28)	0.76	0.308
**Cigarettes smoking status**			
current	1,579 (12.99)	0.76	
former	1,848 (15.20)	0.76	
never	8,727 (71.80)	0.76	0.882
**Alcohol drinking status**			
current	2,770 (22.88)	0.76	
former	1,267 (10.46)	0.76	
never	8,071 (66.66)	0.77	0.244
**BMI**			
Under/Normal weight (<25)	1,791 (14.96)	0.75	
Overweight (25-29.9)	4,072 (34.00)	0.76	
Obese (≤30)	6,112 (51.04)	0.77	<0.001
**Sedentary behavior**			
No	2,380 (20.94)	0.77	
Yes	8,987 (79.06)	0.76	0.052
**Sit hours/day**			
<2	4,784 (46.58)	0.76	
2-3	2,817 (27.43)	0.74	
>3	2,670 (26.00)	0.75	<0.001
**Sleep hours/day**			
<=6	3,307 (28.96)	0.74	
7-8	6,894 (60.36)	0.75	
≥9	1,220 (10.68)	0.77	0.001
**Any Major Disease (prevalence)**			
Yes	7,298 (60.01)	0.77	
No	4,864 (39.99)	0.75	0.013
**High blood pressure (prevalence)**			
Yes	2,372 (19.48)	0.78	
No	9,805 (80.52)	0.76	<0.001
**Diabetes (prevalence)**			
Yes	1,859 (15.27)	0.77	
No	10,317 (84.73)	0.76	0.401
**Gallbladder disease (prevalence)**			
Yes	1,075 (8.83)	0.79	
No	11,102 (91.17)	0.76	0.001
**Asthma (prevalence)**			
Yes	483 (3.97)	0.77	
No	11,694 (96.03)	0.76	0.729
**All cancer (prevalence)**			
Yes	323 (2.65)	0.84	
No	11,855 (97.35)	0.76	<0.001
**Kidney disease (prevalence)**			
Yes	232 (1.91)	0.82	
No	11,945 (98.09)	0.76	0.014

* Adjust for age and gender as appropriate

The mean and median of RTLs were 0.76 and 0.75, respectively. The range was between 0.21 and 0.99. Among demographic characteristics, the most significant association with RTL was observed for age. As expected, we observed a significant trend of decreasing RTL with increasing age categories (*p* < 0.001). Study subjects who had less than high school education had marginally shorter RTL than those who had at least high school education (*p* = 0.069). Study subjects with self-insurance had significantly longer RTL than those without (*p* = 0.004). No significant associations were observed with immigration related variables (including birth location, acculturation, years lived in U.S., and age at arrival).

**Figure 1:**
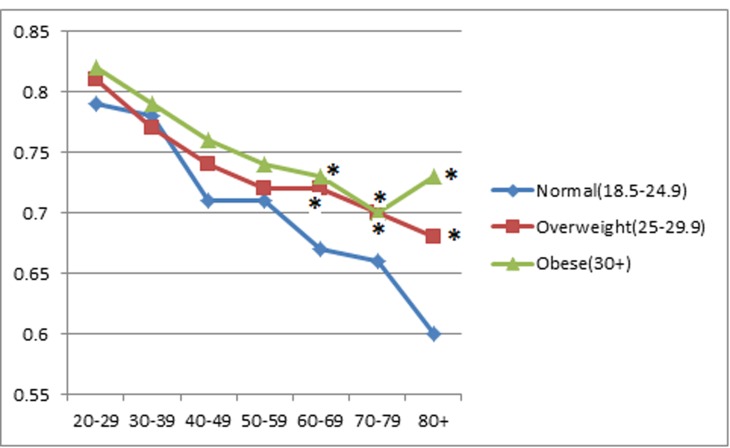
Associations between relative telomere length and age group by BMI category * indicates statistical significance when RTL in overweight or obese subjects were compared to RTL in subjects with a normal weight in each age group.

BMI was positively associated with RTL (both as continuous variables) (*p* < 0.001). In the BMI categorical analysis, after adjusting for age and gender, RTL was shortest among study subjects who were under or normal weight (0.75), median among those who were overweight (0.76), and longest among those who were obese (0.77) (*p* < 0.001). When the study subjects were stratified by age categories, 20-29, 30-39, 40-49, 50-59, 60-69, 70-79, and 80 or more years old (Figure 1), the positive association between RTL and BMI persisted for most of the age categories. However, the BMI category difference in RTL tended to decrease with increasing age. When we compared RTL between the 20-29 years old and those aged 80 or older, the difference was 0.19 for those with under or normal weight, 0.13 for those overweight, and 0.085 for obese groups (*p* = 0.005).

Significant relationships were also observed for RTL with sitting time and sleeping time. Study subjects who had less than 2 hours sitting time per day had the longest RTL (0.76) compared to those who had 2-3 hours (0.74) and more than 3 hours sitting time per day (0.75) (*P* < 0.001). RTL was positively associated with hours of sleeping per day. RTL was the longest among those who slept at least 9 hours per day (0.77), median among those who slept 7-8 hours per day (0.75), and shortest among those who slept no more than 6 hours per day (0.74) (*P* < 0.001). In addition, those who were sedentary had marginally shorter RTL than those who were not sedentary (0.76 *vs* 0.77, *P* = 0.052).

For the six chronic diseases (including hypertension, type-2 diabetes, gallbladder disease, asthma, cancer, and kidney disease) reported at baseline, study subjects who had at least one of the six diseases had longer RTL than those who did not (RTL: 0.77 *vs* 0.75, *p* = 0.013). When stratified by the type of chronic disease, significant difference remained for hypertension (*P* < 0.001), gallbladder disease (*P* < 0.001), cancer (*P* < 0.001), and kidney disease (*P* = 0.014). Using the mediation analysis, we examined the potential mediating role of RTL on the association between BMI and major chronic diseases. RTL was found to mediate 5.25% of the association between BMI and hypertension and 5.13% of the association between BMI and gallbladder disease (*P* = 0.001 and 0.037, respectively).

All social-demographic and behavioral variables with p value ≤ 0.25 in the univariate analysis (from Table 1) were included into a multivariate model (Table 2). After multivariate adjustment, a number of variables remained significantly related to RTL. Longer RTL was positively associated with BMI, education, hours of sleep, and self-insurance, but was inversely associated with age and sitting time.

**Table 2:** Multivariate regression analysis of RTL on social-demographics and health behaviors

Variables	Estimate	Standard Error	*P* value
Age	-0.003	0.0002	<0.001
Gender (women vs men)	0.002	0.008	0.804
BMI	0.002	0.0005	<0.001
Education (≥ high school vs < high school)	0.013	0.006	0.021
Birth place (U.S. vs Mexico)	0.009	0.007	0.197
Ever vs never smokers	-0.008	0.007	0.230
Ever vs never drinkers	0.009	0.007	0.220
Sleep time (≤6 hours/day)			
7-8 hours/day	0.028	0.009	0.003
≥9hours/day	0.039	0.010	0.001
Sitting time (<2 hours/day)			
2-3 hours/day	-0.003	0.008	0.674
>3 hours/day	-0.026	0.007	<0.001
Sedentary lifestyle (yes vs no)	-0.0003	0.007	0.965
Self-insurance (yes vs no)	0.012	0.006	0.041

**Figure 2:**
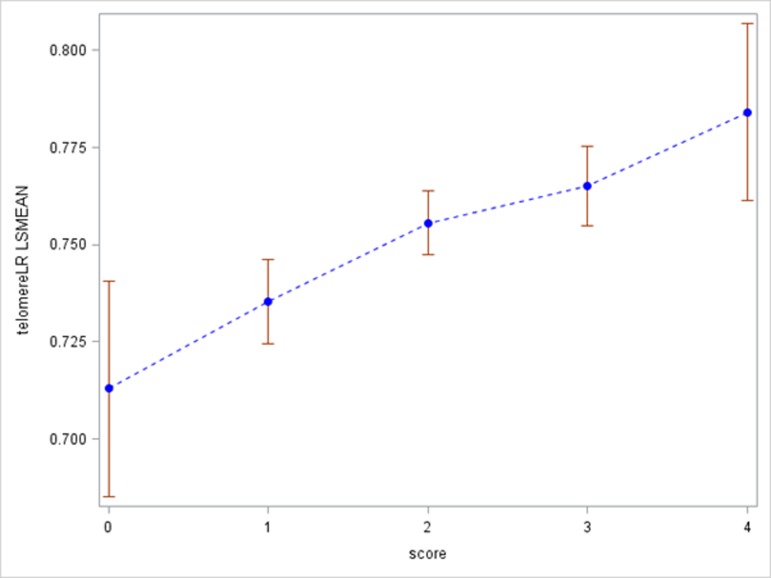
Associations between relative telomere length and social-behavioral score

To assess the potential joint effect of SES and health behaviors on RTL, we created a social-behavioral score by including 2 SES related variables (education and self-insurance) and 2 lifestyle behaviors (sitting time and sleeping time). For each variable, we created a binary high SES or healthy variable, defined as at least high school education, having self-insurance, sitting less than 2 hours per day, or sleeping more than 6 hours per day. The social-behavioral score ranged from 0 (low SES and no healthy behaviors) to 4 (high SES and healthy behaviors). We investigated the association between the social-behavioral score and RTL (Figure 2). In general, when the score increased from 0 to 4, RTL gradually increased (*p* < 0.001). For study subjects with social-behavioral score 0, 1, 2, 3, and 4, RTL was 0.71, 0.73, 0.75, 0.77, and 0.78, respectively.

To assess whether RTL was a predictor of chronic diseases, we analyzed the relationships between RTL and incident cancer and type-2 diabetes during the follow-up. Telomere length data were available from 360 incident cancer cases. Overall, RTL at baseline was not significantly different between incident cancer cases and non-cancer cohort participants (0.77 *vs* 0.76, *p* = 0.555). In the multivariable-adjusted Cox proportional-hazard regression model, using RTL as a continuous variable, we found that longer RTL was associated with increased risk of overall cancer (adj.HR = 1.05, 95%CI = 1.02-1.09) (Table 3). When stratified by cancer site, due to small sample size, no significant association was observed for any cancer type. In further quartile analysis, compared to individuals with the shortest RTL (1^st^ quartile), those who had longer RTL (2^nd^, 3^rd^, and 4^th^ quartiles) had increased risks of overall cancer (*P* for tread = 0.006). However, the only significant association was observed among those in the 4^th^ quartile of RTL (adj.HR = 1.53, 95%CI = 1.11-2.10). Telomere length data were available from 533 incident type-2 diabetes cases. Overall, RTL was associated with decreased risk of incident type-2 diabetes (adj.HR = 0.89, 95%CI = 0.82-0.94) (Table 4). In the quartile analysis, those with longest RTL (4^th^ quartile) had 48% decreased risk of developing type-2 diabetes than those with shortest RTL (1^st^ quartile) (adj.HR = 0.52, 95%CI = 0.32-0.70). A significant trend of decreasing risk of type-2 diabetes was observed when RTL increased (*P* for trend < 0.001).

**Table 3:** Risk of incident cancer associated with RTL

RTL (continuous variables)	Cases, N (%)	Controls, N (%)	Adjusted hazard ratios (HRs) (95%) CI)	*P* value
All cancers	360	9,989	1.05 (1.02-1.09)	0.005
Breast cancer (women) only)	87	8,170	1.01 (0.94-1.07)	0.889
Lung cancer	26	10,294	1.11 (0.96-1.27)	0.159
Cervical cancer (women) only)	19	8,225	1.05 (0.92-1.21)	0.454
Liver cancer	11	10,303	1.03 (0.85-1.25)	0.773
Prostate cancer (men)	21	2,049	1.16 (0.97-1.37)	0.097
**All cancers, by quartile**				
1st	92 (25.6%)	2,679 (26.8%)	1.00	
2nd	94 (26.1%)	2,335 (23.4%)	1.17 (0.88-1.57)	0.275
3rd	104 (28.9%)	2,570 (25.7%)	1.31 (0.99-1.73)	0.063
4th	70 (19.4%)	2,405 (24.1%)	1.53 (1.11-2.10)	0.009
P for trend				0.006
			

Adjusted for age, sex, birth country, education, marital status, HBP, diabetes, smoking, drinking, sitting time, and physical activity.

**Table 4:** Risk of incident diabetes associated with RTL

	Diabetes, *N* (%)	Non-diabetes, *N* (%)	HR(95%CI)*	*P* value
All study subjects				
RTL (continuous variables)	533	7,837	0.89 (0.82-0.94)	0.001
By quartile				
1st	195 (36.6%)	2,108 (26.9%)	ref	
2nd	139 (26.1%)	1,834 (23.4%)	0.82 (0.55-1.03)	0.084
3rd	124 (23.2%)	2,006 (25.6%)	0.67 (0.45-0.90)	0.001
4th	75 (14.1%)	1,889 (24.1%)	0.52 (0.32-0.70)	<0.001
P for trend				<0.001

*adjusted for age, sex, birth country, education, marital status, HBP, smoking, drinking, sitting time, and physical activity.

Last, in a subset of study subjects with multiple weight data during follow-up, we explored whether RTL could predict weight gain during the follow-up (Table 5). At least 10% weight gain compared to the baseline weight during the follow-up was deemed as the event. In the multivariate regression analysis, the final model included age at baseline, gender, baseline BMI, marriage status, acculturation, birth place, and RTL. RTL was a positive predictor of at least 10% weight gain during the follow-up (adj.HR = 1.03, 95%CI = 1.00-1.05). In addition, older age, married (or living together), high BMI, and born in U.S. were associated with less likely to gain at least 10% weight. On the other hand, being women and having higher acculturation were more likely to gain at least 10% weight.

**Table 5:** Risk of weight gain (>=10%) and RTL

	HR(95%CI)	*P* value
Age	0.97(0.97-0.98)	<.0001
Gender: men vs women	0.78(0.63-0.97)	0.0229
BMI	0.94(0.94-0.95)	<.0001
marital status: other vs married	1.24(1.08-1.42)	0.0021
acculturation	1.05(1.00-1.12)	0.0743
Own home: yes vs no	0.79(0.71-0.89)	<.0001
Self-insurance: no vs yes	0.84(0.76-0.94)	0.0018
logRTL (0.1 per unit)	1.02(1.01-1.04)	0.0075

## DISCUSSION

The associations among social-demographics, health behaviors, and telomere length have rarely been studied in adult Mexican Americans. The only published study was from the National Health and Nutrition Examination Survey (1999-2002), which includes 1,377 Mexican Americans [49]. In that study, the only significant factor correlated with RTL was age. In the current study, with 12,792 Mexican Americans aged 20 to 85 years, in the cross-sectional analysis, we found that lower SES (measured by low education levels and without self-insurance) was significantly associated with shorter RTL. In addition, we observed RTL was positively correlated with BMI, sleeping time, and several major chronic diseases, and negatively correlated with age and sitting time. Then, in the longitudinal analysis, we found that longer RTL was a predictor for all cancer, type-2 diabetes, and weight gain.

Socioeconomic disparities in chronic diseases are well-known, but we have limited understanding about the biological mechanisms underlying the association between social status and health. Individuals with low SES are exposed to more stressful conditions, such as chronic financial strain and exposure to harmful work and home environments [50]. Low SES is linked with reduced access to psychosocial means, such as self-efficacy and social support, that can buffer the harmful impact of stress on health [50]. Furthermore, given the stress responses are triggered by experiences in which individuals feel that the resources they have at hand are sufficient to deal with a danger, it is expected that individuals with low SES are prone to exhibiting larger dysregulation of stress response systems [51]. Given the growing body of evidence demonstrating an association between telomere length and stressful circumstances, telomere length provides a potential biological link between low SES and chronic diseases. In the current study, we observed an inverse relationship between levels of SES and RTL. Study subjects with less than high school education had shorter RTL than those who had at least high school education (*P* = 0.021). Similarly, study subjects with no self-insurance had shorter RTL than those who had self-insurance (*P* = 0.041). Our results are consistent with several previous studies. For example, using data from the National Health and Nutrition Examination Survey (1999-2002), Needham et al. found that study subjects who completed less than high school education has significantly shorter telomeres than those who graduated from college (*P* < 0.01) [49]. However, the significant relationship was only observed among Caucasian American study subjects, not among African and Mexican Americans. The only significant relationship reported among Mexican Americans was from Multi-Ethnic Study of Atherosclerosis (MESA), which evaluated the relationship between SES and telomere length among 963 U.S. adolescents, including 510 Mexican American adolescents [52]. In that study, Carroll et al. found that father’s education and current home ownership was associated with telomere length.

Health behavior is a major determinant of chronic disease risk in most of the developed countries [19, 20, 24, 27]. Epidemiological and basic science studies have shown that unhealthy lifestyle including cigarette smoking, heavy drinking, sedentary behavior, and obesity could increase stress levels, decrease immune function, and deteriorate physiological condition [53-58]. In the current study, we found inverse relationships among sitting time, sleeping time, and RTL, and a positive relationship between BMI and RTL. No significant relationship was observed among cigarette smoking, alcohol drinking, sedentary behavior, and RTL. The relationship among sedentary behavior, sitting time, and RTL has been investigated previously [31, 49, 59-66]. The results are still limited and inconsistent. For example, Sjögren et al. found that shorter sitting time was associated with telomere lengthening in blood cells in sedentary, overweight 68-year-old individuals participating in a 6-month physical activity intervention trial [66]. Using data from Nurses’ Health Study, Du et al. reported that sitting time and sedentary behavior were not associated with leukocyte telomere length [31]. However, they found that moderate to vigorous amounts of physical activity are associated with longer telomeres. Much of the discrepancy among different studies may be due to the different methods used to assess physical activity, sedentary behavior, and sitting time. Differences in study population, such as race, age, occupation, and sample size etc., may also contribute to the inconsistent results. Telomere length has been reported to be more stable among older individuals [67], and weakened associations have been witnessed among older individuals [61], potentially reducing the statistical power in studies of older participants. Compared to many of the other studies, our study population is younger (mean age = 42 years old). Coupled with the large sample size (*N* = 12,792), our study is more powerful to detect significant associations.

One interesting finding in this study is the positive relationship between sleeping time and RTL. The relationship between sleep and telomere length has been investigated in a few population-based studies [68-72]. In a study of participants in the Nurses’ Health Study, short sleep duration (≤6 hours) was associated with shorter telomere length only in those younger than 50 years [71]. Jackowska et al. found a positive linear relationship between sleep time and leukocyte telomere length in men only [69]. Thus, our results confirmed the previous reports and further suggest the positive relation between sleeping time and telomere length is consistent regardless of gender. The mechanisms through which sleep might be related to telomere length remain to be determined, although several molecular pathways have been suggested, including inflammation [73, 74], oxidative stress [75], sympathetic nervous system activity [76, 77], and neuroendocrine pathways [78, 79].

Another intriguing but puzzling finding from this study is the positive relationship between BMI and telomere length. In a recent systematic review and meta-analysis of 29 existing studies, Muezzinler et al. concluded an inverse association between BMI and RTL in adults [80]. However, in the Genetic Epidemiology Research Study on Adult Health and Aging (GERA) with 100,000 study subjects presented in 2012 American Society of Human Genetic Annual meeting, Schaefer et al. reported that BMI was positively correlated with telomere length in saliva DNAs, which is consistent with our finding. To eliminate the potential age effect, we stratified the study subjects based on their age categories, and we found study subjects in the obese group had steadily longer telomere length than those in the normal weight group across all the age categories. More interestingly, the difference of telomere length by BMI status became larger with the age increased. In studying the relationship between RTL and prospective weight gain, we found that long RTL at baseline was associated with a higher likelihood of gaining at least 10% weight during follow-up. Our observation is consistent with the results from the Health ABC study, in which longer telomere length was associated with a positive percent change between baseline and 7-year follow-up for both BMI and % body fat [81]. Clearly, more work is needed to determine the underlying molecular mechanisms between BMI and RTL.

The relationship between RTL and major chronic diseases (both prevalent and incident) was another piece of interesting finding. At the cross-sectional comparison, longer RTL was associated with increased prevalence of overall major chronic diseases as well as several diseases individually, including hypertension, gallbladder disease, cancer, and kidney disease. Prospectively, longer RTL was associated with an increased risk of overall cancer. The positive association seems consistent across the cancer type. However, no significant association was observed in any individual type of cancer, likely due to the small sample size in each type of cancer. The relationship between RTL and cancer has been studied extensively in both case control and cohort settings [9, 82-98]. However, the results are inconsistent at large. Recent large cross-sectional studies and prospective studies have shown that both long RTL and short RTL can predispose individuals to increased cancer risks and the direction of association is not only cancer type-dependent, but also histology-dependent [93, 96, 98]. For example, long RTL was associated with an increased risk of lung adenocarcinoma, whereas short RTL was associated with a reduced risk of lung squamous cell carcinoma [93, 98]. Although we do not know the exact biological explanation, upon exposure to carcinogens, cells with longer telomeres may be less likely to enter senescence or apoptosis. Their survival could increase their chance to have extended carcinogen exposure and thereby increase the risk of developing genetic abnormalities.

In a recent meta-analysis including 4 cohort studies and 4 case-control studies, Zhao et al. found that shortened telomere length was significantly associated with risk of type-2 diabetes (OR = 1.29, 95% CI: 1.11-1.14) [10]. Similar association was observed in our study. Short telomeres may lead to premature β-cell senescence, resulting in reduced β-cell mass and subsequent impaired insulin secretion and glucose tolerance. Indeed, experimental evidence suggests that telomerase is important in maintaining glucose homeostasis in mice [99]. Conversely, elevated blood glucose levels increase oxidative stress, potentially interfering with telomerase function and resulting in shortened telomeres [100].

There are several limitations in this study. First, the measure of RTL used in this study is an average of telomere length across all leukocyte cell types. Previous research suggests that telomere length in different cell types varies within the same individual [101]. Future work should attempt to evaluate RTL in a single cell type. Second, the numbers of incident cancer and diabetes are still small, especially for cancer. This limits our statistical power to detect the associations. Thus, our data need to be interpreted with caution. Third, the analysis of SES and health behavior in relation to RTL is cross-sectional. Longitudinal data would be necessary to analyze the rate of telomere shortening in relation to baseline SES and health behavior. Finally, we did not have a direct assessment of stress exposure and cannot estimate the extent to which SES and health behavior affect RTL through stress physiology.

The relationships among SES, health behavior, chronic diseases and telomere length have been studied previously [2, 19]. However, such study has never been comprehensively investigated among adult Mexican Americans. This study fills the gap. Among 12,792 Mexican Americans, we found that RTL is significantly affected by SES and health behavior. In addition, we found longer RTL is a predictor of cancer, diabetes, and weight gain. Together with the results of other studies in different populations and different races, this study suggests that the investigation of telomere length can help further our understanding on how social conditions and health behavior can transduce into molecular pathways and eventually affect health.

## MATERIALS AND METHODS

### Study population

The samples for the current study were drawn from participants in a large population-based cohort of Mexican origin households recruited from the Houston-area. This cohort, an ongoing prospective cohort of 1^st^ and 2^nd^ generation Mexican origin immigrant households in Houston, TX, initiated in July 2001 and maintained by Department of Epidemiology at the University of Texas MD Anderson Cancer Center in Houston, Texas. A detailed description of the sampling and recruitment strategy has been published previously [102]. Briefly, participants have been recruited through block walking in predominantly Mexican American neighborhoods, from community centers and local health clinics, and networking through currently enrolled participants. Of the identified eligible households, ∼88% agreed to participate in the study. After written informed consent was obtained, trained bilingual research interviewers conducted a structured face-to-face interview lasting ∼45 minutes, using a standardized and validated questionnaire in the participant’s preferred language, either Spanish or English. The questionnaire elicited information on birthplace and residential history, social-demographic characteristics, lifestyle behaviors, levels of physical activity, personal medical history, family history of chronic disease, acculturation, and occupation exposure. The study was approved by the institutional review board of M.D. Anderson Cancer Center. Participants have been actively followed up *via* annual telephone re-contact to update selected exposures and new diagnosis of selected chronic diseases, including cancer, type-2 diabetes, and hypertension. The incidence of cancer was confirmed with Texas Cancer Registry and the incidence of type-2 diabetes was confirmed by medical record review.

### Relative telomere length (RTL) assessment

Genomic DNA was isolated from whole blood samples of 12,792 participants using the QIAamp Maxi DNA kit (Qiagen, Valencia, CA) according to the manufacturer’s protocol. Extracted DNA samples were quantified by Quant-iT^™^ PicoGreen^®^dsDNA Reagent and Kits (Invitrogen, Carlsbad, CA) according to the manufacturer’s directions. The RTL was measured using a modified version of the real-time quantitative polymerase chain reaction (PCR) method originally described by Cawthon [103]. A detailed description of the experimental strategy has been published previously [104]. Briefly, the ratio of the telomere repeat copy number (T) to the single gene (human globulin) copy number (S) was determined for each sample. The derived T/S ratio was proportional to the overall telomere length. The PCR reaction mixture (14 ul) for the telomere amplification consisted of 1 x SYBR Green Master Mix (Applied Biosystems, Foster City, CA), 200 nmol/l Tel-1 primer (5’-CGGTT TGTTTGGGTTTGGGTTTGGGTTTGGGTTTGGGTT), 200 nmol/l Tel-2 primer (5’-GGCTT GCCTTACCCTTACCCTTACCCTTACCCTTACCCT), and 5 ng genomic DNA. Similarly, the PCR reaction mixture (14 ul) for *HGB* gene amplification consisted of 1x SYBR Green Master Mix, 200 nmol/l Hgb-1 primer (5’-GCTTCTGACACAACTGTGTTCACTAGC), 200 nmol/l Hgb-2 primer (5’-CACCAA CTTCATCCACGTTCACC), and 5 ng genomic DNA. The thermal cycling conditions were 1 cycle at 95°C for 10 minutes followed by 40 cycles at 95°C for 15 seconds and at 56°C (for telomere amplification) or 58°C (for *HGB* amplification) for 1 minute. The HT7900 system (Applied Biosystems, CA) was used to perform the Real-time PCR. Each sample was run in duplicate in a 384-well plate. The telomere and *HGB* PCRs were done on separate 384-well plates with the same samples in the same well positions. In each run, negative and positive controls, a calibrator DNA, and a standard curve were included. The positive controls contained a telomere of 1.2 kb and a telomere of 3.9 kb from a commercial telomere length assay kit (Roche Applied Science, Pleasanton, CA). For each standard curve, 1 reference DNA sample (the same DNA sample for all runs) was diluted 2-fold serially to produce a 6 point standard curve between 20 ng and 0.625 ng of DNA in each reaction. The coefficient of determination (R^2^) for each standard curve was≥0.99, with acceptable standard deviations set at 0.25 (for the Ct values). The intra-assay coefficient of variation was < 3%, and the inter assay coefficient of variation was < 5% for RTL assay in our laboratory.

### Statistical analysis

We used the statistical software package SAS, version 9.4 (SAS, Cary, NC) for analysis. Since the relative telomere length data were not normally distributed, we performed the analysis using both data with and without log transformation. We found no significant differences in the estimated associations with and without log transformation, and therefore only data without log transformation were presented here. Differences in the distribution of social-demographics, health behaviors, and major chronic diseases were evaluated by Pearson χ^2^ test for categorical variables. Spearman correlation was used to assess the relationship between age and RTL. In univariate analysis, the general linear regression analysis was used to examine RTL by the selected variable. Both age and gender were adjusted as appropriate. Mediation analysis was performed to assess the role of RTL in the association between BMI and chronic diseases at baseline. In multivariate analysis, the general linear model was used. The social-demographic and health behavior variables associated with RTL with a P value less than 0.25 in the univariate analysis were included in the multivariate analysis. For joint effect analysis, we created a social-behavioral score by including 2 SES related variables (education and self-insurance) and 2 lifestyle behaviors (sitting time and sleeping time). These are binary variables with the high SES or healthy category defined as at least high school education, having self-insurance, sitting less than 2 hours per day, and sleeping more than 6 hours per day, respectively. The social-behavioral score ranged from 0 (low SES and no healthy behaviors) to 4 (high SES and healthy behaviors). Multivariate analysis was used to assess the relationship between the social-behavioral score and RTL. The associations of incident cancer and type-2 diabetes with RTL were assessed using the multivariable-adjusted Cox proportional-hazard regression model. RTL was analyzed as both a continuous and categorical variable. For categorical variables, cutoff points were set at the quartile values in the non-cancer study population. Adjusted Hazard ratios (HR) and 95% confidence intervals (CI) were estimated and potential confounding factors were adjusted as appropriate. Multivariable-adjusted Cox proportional-hazard regression model was also used to examine whether RTL at baseline could predict at least 10% weight gain in the follow-up. All statistical tests were 2-sided, and the level of statistical significance was set at 0.05.
